# Takotsubo syndrome: left atrial and ventricular myocardial strain impairment in the subacute and convalescent phases assessed by CMR

**DOI:** 10.1186/s41747-024-00423-7

**Published:** 2024-02-28

**Authors:** Giacomo Pambianchi, Livia Marchitelli, Giulia Cundari, Letizia Ruoli, Luca Conia, Carlo Catalano, Nicola Galea

**Affiliations:** Department of Radiological, Oncological and Pathological Sciences, Sapienza University of Rome — Policlinico Umberto I Hospital, Viale Regina Elena 324, Rome, 00183 Italy

**Keywords:** Atrial function, Cardiac magnetic resonance, Female, Takotsubo cardiomyopathy, Ventricular function

## Abstract

**Background:**

We investigated the differences in impairment of left ventricle (LV) and left atrium (LA) contractile dysfunction between subacute and convalescent takotsubo syndrome (TTS), using myocardial strain analysis by cardiac magnetic resonance (CMR) feature-tracking technique.

**Methods:**

We retrospectively selected 50 patients with TTS clinical-radiological diagnosis who underwent CMR within 30 days since symptoms onset: 19 studied during the early subacute phase (sTTS, ≤ 7 days) and 31 during the convalescence (cTTS, 8–30 days). We measured the following: LV global longitudinal, circumferential, and radial strain (lvGLS, lvGCS, lvGRS) and strain rate (SR) and LA reservoir (laS_r), conduit (laS_cd), and booster pump strain (laS_bp) and strain rate (laSR_r, laSR_cd, laSR_bp). Patients were compared with 30 age- and sex-matched controls.

**Results:**

All patients were women (mean age 63 years). TTS patients showed altered LV- and LA-strain features, compared to controls. sTTS was associated with increased laS_bp (12.7% *versus* 9.8%) and reduced lvEF (47.4% *versus* 54.8%), lvGLS (-12.2% *versus* 14.6%), and laS_cd (7.0% *versus* 9.5%) compared to cTTS (*p* ≤ 0.029). The interval between symptoms onset and CMR was correlated with laS_bp (*r* = -0.49) and lvGLS (*r* = 0.47) (*p* = 0.001 for both). At receiver operating characteristics analysis, laS_bp was the best discriminator between sTTS and cTTS (area under the curve [AUC] 0.815), followed by lvGLS (AUC 0.670).

**Conclusions:**

LA dysfunction persists during the subacute and convalescence of TTS. laS_bp increases in subacute phase with progressive decrease during convalescence, representing a compensatory mechanism of LV dysfunction and thus a useful index of functional recovery.

**Relevance statement:**

Atrial strain has the potential to enhance the delineation of cardiac injury and functional impairment in TTS patients, assisting in the identification of individuals at higher risk and facilitating the implementation of more targeted and personalized medical therapies.

**Key points:**

• In TTS, after ventricular recovery, atrial dysfunction persists assessable with CMR feature tracking.

• Quantitative assessment of atrial strain discriminates atrial functions: reservoir, conduit, and booster pump.

• Atrial booster pump changes after acute TTS, regardless of ventricular function.

• Atrial strain may serve as a temporal marker in TTS.

**Graphical Abstract:**

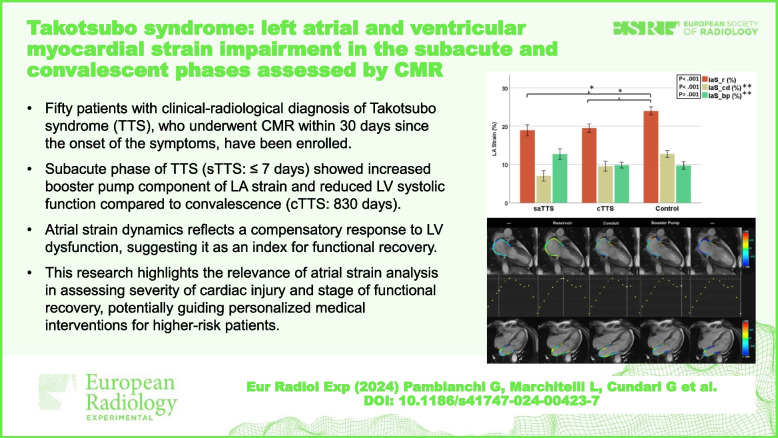

**Supplementary Information:**

The online version contains supplementary material available at 10.1186/s41747-024-00423-7.

## Background

Takotsubo syndrome (TTS), also known as “stress cardiomyopathy,” is a condition presenting as an acute coronary syndrome with transient ventricular systolic dysfunction, without the obstruction of coronary arteries, often triggered by emotional or physical stressors [1].

The pathophysiological mechanisms are still not fully clarified, even though the massive release of catecholamines such as adrenaline and norepinephrine during acute stress is thought to play a key role [2].

The main imaging feature of TTS is the presence of a regional contractile dysfunction of the left ventricular (LV) wall, typically involving the apical segments, determining the peculiar circumferential systolic enlargement of the LV apex, named “apical ballooning” [3, 4].

TTS diagnosis may be challenging, due to the wide variety of symptoms and atypical patterns [3], and relies on multiple criteria such as the revised Mayo Clinic criteria [3], the Heart Failure Association-European Society of Cardiology Criteria [4], and the International Takotsubo Diagnostic Criteria [5]. The prevalence of TTSs has steadily increased over the years, now accounting for 0.7 to 2.2% of patients and 5 to 6% of women with suspected acute coronary syndrome [2]. Based on published literature, about 90% of TTS patients are women with an average age of 67–70 years and about 80% over 50 years [6], with a predilection for postmenopausal women. Although considered a benign and self-limiting condition, recent evidence has shown that TTS patients may experience a persistent cardiac dysfunction [7] and symptoms, despite the recovery of LV ejection fraction (EF) [8].

Cardiac magnetic resonance (CMR) has emerged as the reference standard [9] for TTS diagnosis in the acute-subacute phase, differentiating with high accuracy TTS from other acute cardiac conditions with similar clinical presentation (*e.g.*, myocarditis or myocardial infarction without coronary obstruction) [10, 11]. Indeed, CMR enables a combined assessment of morphology and function and facilitates the detection of myocardial edema on T2-weighted images, as well as the identification of myocardial scarring by late gadolinium-enhanced (LGE) imaging [10, 12]. Additionally, CMR feature tracking is a reliable and useful technique [13] to analyze the ventricular and atrial function on cine images, measuring the wall deformability (myocardial strain) [14]. CMR feature-tracking analysis enables to characterize the impairment of the different components of atrial function (reservoir, conduit, and booster pump), as demonstrated in various cardiac pathologies, including dilatative cardiomyopathy, myocardial infarction, and hypertrophic cardiomyopathy [15–17].

Left atrial (LA) transient impairment during TTS has been already described [18] and could play a role in the prognostic stratification for adverse events [19]. However, still limited data regarding the atrial involvement in TTS are available [20, 21]. In particular, the modification of atrial function during the subacute and early convalescent phases needs to be clarified. Thus, the aim of the study was to characterize the LA and LV contractile dysfunction in TTS patients during the subacute and convalescent phases and to investigate the potential role of atrial strain features in discriminating the different phases of TTS.

## Methods

### Study population

The study was conducted in accordance with the Declaration of Helsinki. Approval of the ethical committee was obtained, and all participants gave written informed consent to participate in the study, after signing a general informed consent for the use of their data for research purposes.

Among patients admitted to the emergency department between January 2015 and May 2023, with a diagnosis of TTS based on Mayo Clinic criteria [4], we retrospectively evaluated only patients who underwent CMR examination within 30 days from the onset of symptoms. Each patient presented all the following features:

1. Acute chest pain and/or dyspnea
2. New electrocardiographic abnormalities (either ST elevation or T-wave inversion) and cardiac troponin elevation
3. Ventricular dysfunction at echocardiography performed within 24 h from admission
4. The absence of obstructive coronary artery disease at invasive or computed tomography coronary angiography

We excluded patients with the following:

1. Insufficient image quality due to the presence of extensive artifacts or incomplete atrial representation
2. Previous known cardiac disease
3. Moderate-to-severe mitral valve regurgitation
4. Atrial fibrillation

A control group of 30 age- and sex-matched patients, without known cardiac diseases, who underwent CMR for other indications revealing normal ventricular size and function and the absence of any myocardial signal abnormalities was retrospectively enrolled.

### CMR protocol

Standard CMR examinations were performed on a 1.5-T unit (MAGNETOM Avanto, Siemens Healthineers, Erlangen, Germany), using body and eight-channel-phased array coils. Breath-hold steady-state free-precession cine (cineMR) and black-blood T2-weighted short tau inversion-recovery (T2-STIR) sequences were acquired on cardiac long- and short-axes views with full coverage of both ventricles. LGE images were acquired by late postcontrast images, acquired in long-axis and short-axis views, 10–15 min following intravenous administration of a bolus of 0.15 mmol/kg gadobutrol (Gadovist; Bayer Healthcare, Berlin, Germany). Detailed parameters of the sequences are available in our previous report [22].

### Image analysis

The image analysis was conducted by two experienced radiologists (G.P., 7 years of experience, and N.G., 16 years of experience) in consensus, utilizing a dedicated postprocessing software (Cvi42 v5.14, Circle Cardiovascular Imaging, Calgary, AB, Canada).

Atrial and ventricular volumes, along with derived parameters, were measured using cineMR images. Epicardial and endocardial LV borders were traced on the short- and long-axis images in a semiautomatic fashion, and body surface area (BSA) was used to index the parameters [23]. T2-STIR and LGE images were evaluated to detect the presence of myocardial edema and fibrosis, respectively, as areas of increase in signal intensity compared to remote myocardium, as previously described [23].

CMR feature-tracking analysis of LV strain was performed using ventricular short- and long-axis views in cineMR images in a semiautomatic way. LV myocardial tracking was visually reviewed, contouring errors were corrected, and the analysis was repeated as previously described [24, 25]. We finally reported the average value of three repeated measurements of global radial (GR-), circumferential (GC-), and longitudinal (GL-) strain (-S) and strain rate (-SR).

**Fig. 1:**
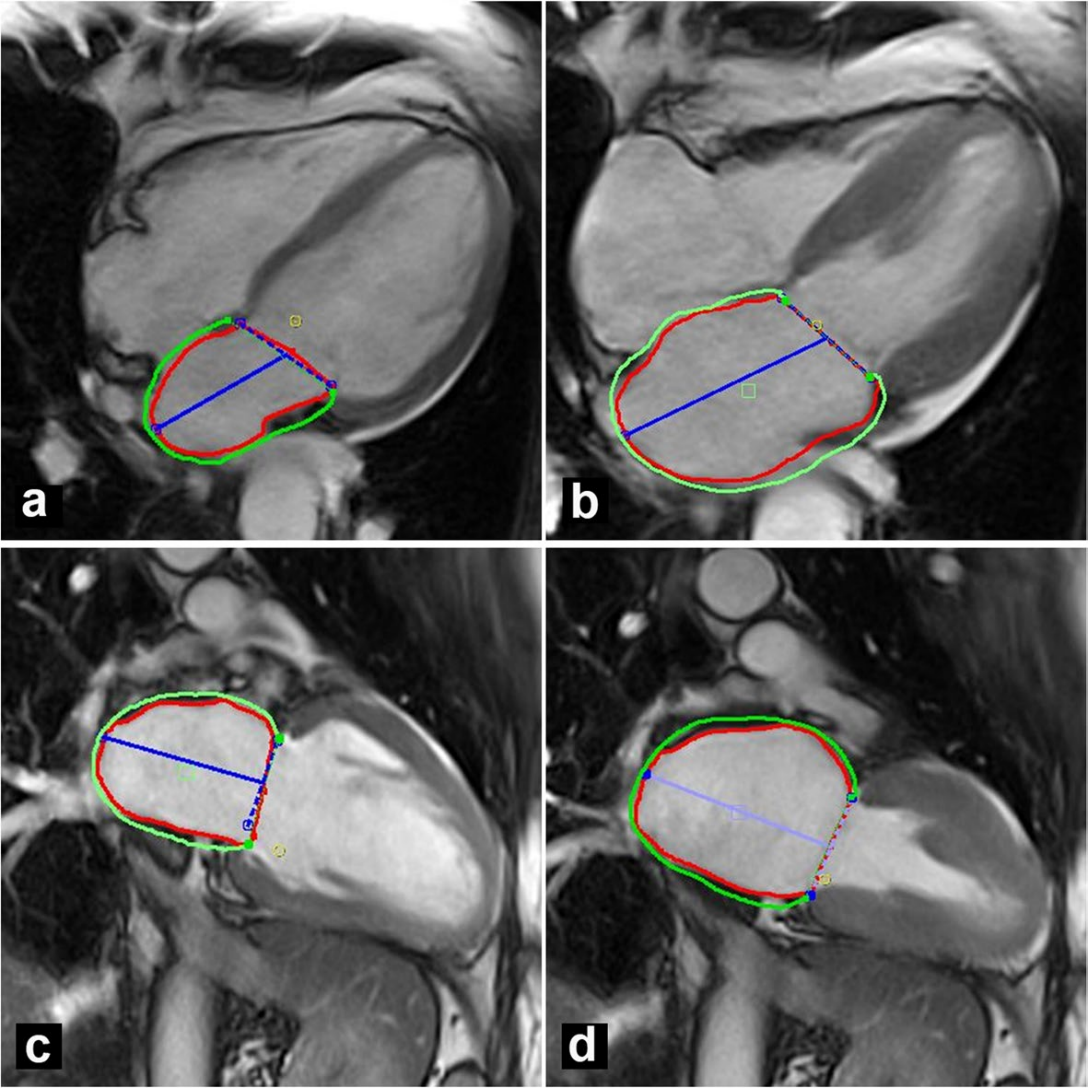
cTTS patient. Four chambers (**a**, **b**) and two chambers (**c**, **d**) long-axis cineMR images. Endocardial (red line) and epicardial (green line) contours of the left atrium in the atrial end-systolic (**a**, **c**) and end-diastolic (**b**, **d**) phases

LA strain analysis was performed using the CMR feature-tracking technique according to the previously reported technique [14]. We traced endocardial atrial borders in cineMR images in horizontal and vertical long-axis images, at the frame point after atrial contraction, and automatically propagated to all other phases (Fig. 1). All the resulting contours were reviewed, corrected if necessary, and validated by operators.

**Fig. 2:**
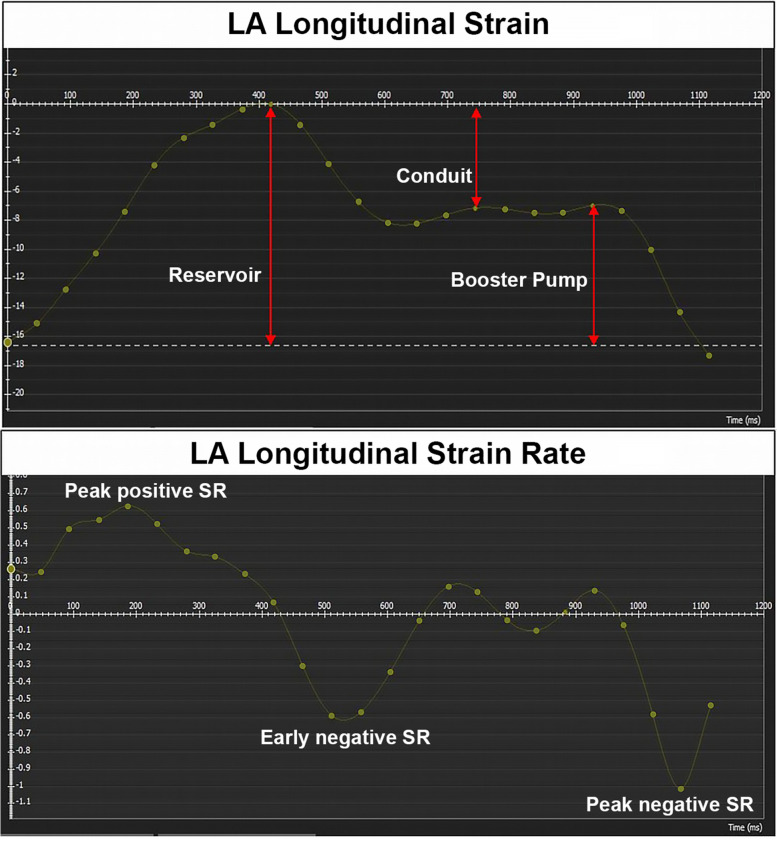
cTTS patient. Left atrial strain (**a**) and strain rate (**b**) curves during a cardiac cycle

**Fig. 3:**
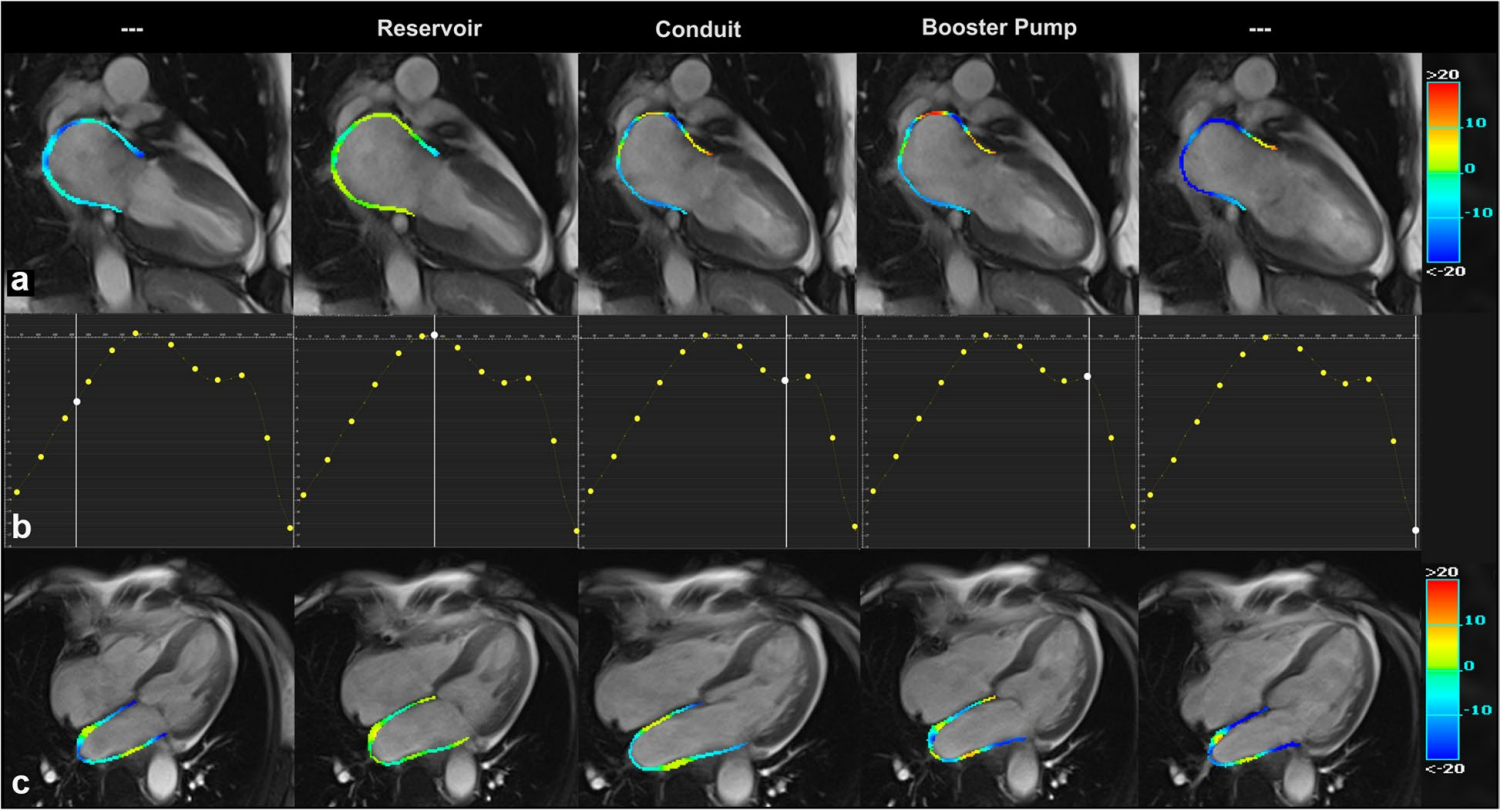
Feature-tracked colorimetric maps of the left atrial longitudinal strain in a subacute takotsubo syndrome patient, superimposed on the cineMR images on vertical (**a**) and horizontal (**c**) long-axis views and respective strain-to-time curves (**b**)

LA reservoir, conduit, and booster pump functions were assessed by measuring longitudinal reservoir strain (laS_r), peak positive strain rate (laSR_r), conduit strain (laS_cd), peak early negative strain rate (laSR_cd), active booster pump strain (laS_bp), and peak late negative strain rate (laSR_bp). All these values were individuated in the corresponding GLS/GLSR-to-time graphs for each patient (Figs. 2 and 3). The LA global radial strain (laGRS) and longitudinal strain (laGLS) were automatically calculated by the software. The entire procedure was repeated three times, and the average values were then reported.

Interobserver and intraobserver variability of LA strain and strain rate was assessed in 25 and 10 subjects, respectively.

### Statistical analysis

Data are presented as counts and percentages for categorical data and mean with standard deviation for continuous parameters. The normal distribution of all variables was tested using Kolmogorov–Smirnov and Shapiro–Wilk tests. Non-normally distributed variables were reported as median with the interquartile range, and independent samples were compared using Mann–Whitney *U* and Kruskal–Wallis tests. A *t*-test for independent samples was applied to evaluate the relationship between continuous variables and to compare the means of the groups. Comparisons between the groups were performed using one-way ANOVA and Bonferroni post hoc analysis for the normally distributed variables.

*χ*^2^ test was performed for the assessment of dependency between two categorical variables. We analyzed the correlation between parameters using Spearman (not normally distributed) and Pearson coefficients for the normally distributed (poor, 0; slight, 0.01–0.20; fair, 0.21–0.40; moderate, 0.41–0.60; good, 0.61–0.80; and excellent, 0.81–1.00).

The intraclass correlation coefficient (ICC) was used to evaluate the intra- and inter-observer variability (ICC, < 0.40, poor; ICC > 0.40–0.75, fair to good; and ICC > 0.75, excellent agreement) for measuring reproducibility of normally distributed variables. To evaluate the correspondence between strain and onset-to-CMR, a linear regression model was carried out. A receiver operator characteristics (ROC) curve was used to determine the diagnostic accuracy for atrial and ventricular strain parameters in differentiating sTTS from cTTS. Youden’s test was applied to identify the optimal strain cut-off values.

Analysis was performed using SPSS (version 27.0, Statistical Package for the Social Sciences, International Business Machines, Inc., Armonk, New York, USA);* p*-values were considered significant if < 0.05.

## Results

### Patient characteristics

**Fig. 4:**
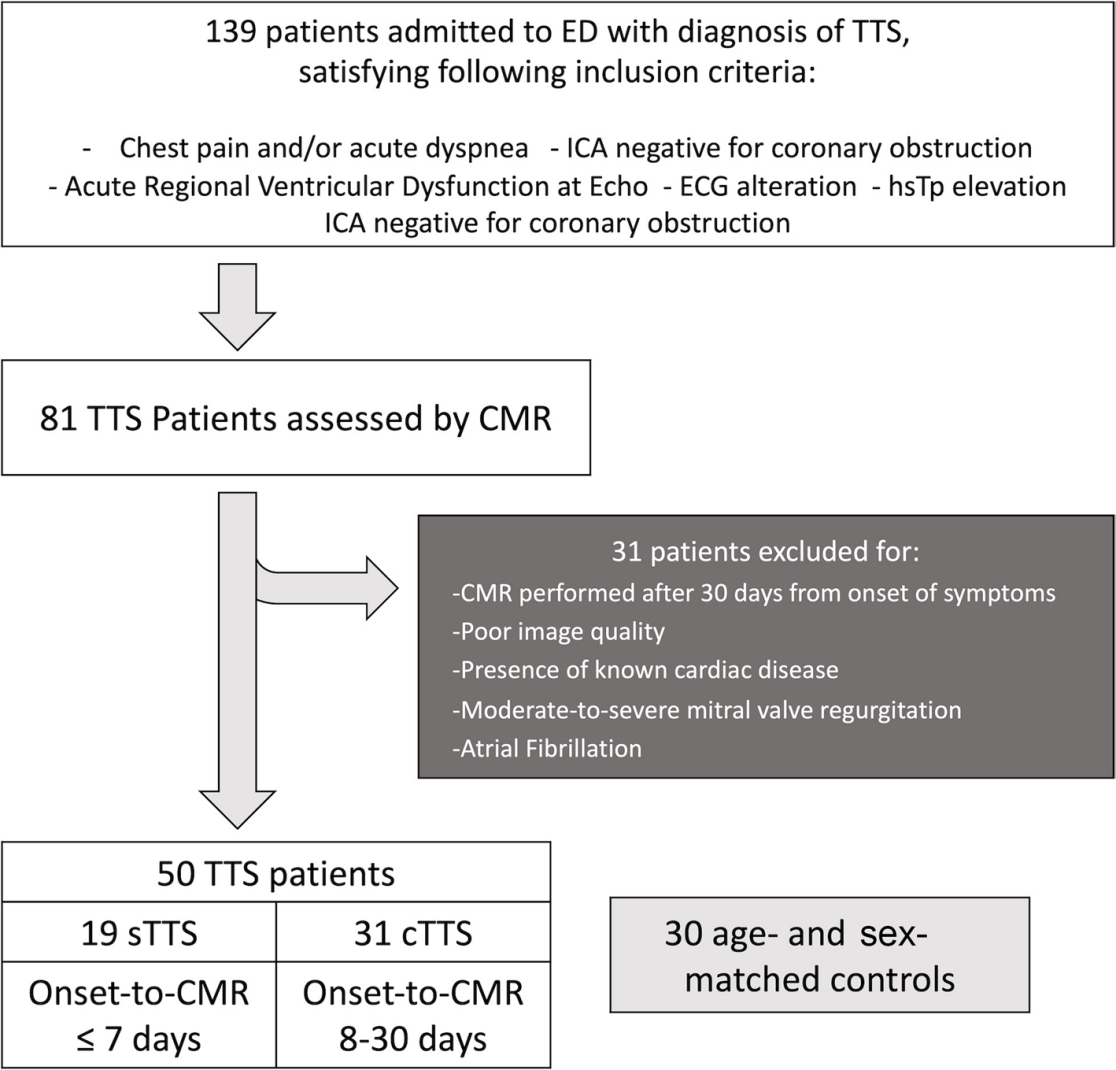
Patient’s recruitment flowchart. *cTTS* Convalescent TTS, *ED* Emergency department, *hsTp* High-sensitivity troponin, *ICA* Invasive coronary angiography, *onset-to-CMR* Time passed between the onset of symptoms and CMR exam, *sTTS* Subacute TTS, *TTS* Takotsubo syndrome

Fifty TTS patients were finally included in the study (Fig. 4), all women, aged 68.5 ± 12.9 years (mean ± standard deviation) with an interval between symptom onset and CMR of 11 ± 7 days. Demographics and clinical data are shown in Table 1. Based on previous studies in the literature investigating the temporal evolution of TTS [26, 27], we divided TTS patients into two categories, according to the time between the onset of symptoms and CMR examination. Therefore, patients were classified as “subacute” (sTTS) if CMR was performed within 7 days (5 ± 2 days), and “convalescent” (cTTS), with an onset-to-CMR time ranging from 8 to 30 days (14 ± 6 days). Clinical and CMR parameters are reported in Table 2.

**Table 1:** Clinical data and CMR parameters of the study population (TTS and controls)

Parameters		TTS	Controls	*p*-value
Population, n		50	30	−
Age, years		69 ± 13	63 ± 11	0.354
Sex, females, n (%)		50 (100)	30 (100)	1.000
Body mass index, kg/m^2^		25.7 ± 5.6	26.2 ± 4.7	0.547
Hypertension, n (%)		16 (32)	10 (33)	0.273
Hyperlipidemia, n (%)		9 (18)	6 (20)	0.588
Diabetes, n (%)		2 (4)	3 (10)	0.612
Smoke, n (%)		8 (16)	7 (23)	0.595
Ex-smoker, n (%)		2 (4)	4 (13)	0.148
Onset-to-CMR, days		11.2 ± 6.5	−	−
Phenotype, n (%)	Apical	36 (72)	−	−
	Mid	8 (16)	−	−
	Basal	2 (4)	−	−
	Focal	4 (8)	−	−
lvEF range, n (%)	< 40%	6 (12)	−	−
	40–50%	10 (20)	−	−
	51–60%	21 (42)	8 (26)	0.127
	> 60%	13 (26)	23 (74)	0.001
lvESV/BSA, mL/m^2^		35.6 ± 15	23.9 ± 9.6	< 0.001
lvEDV/BSA, mL/m^2^		74.6 ± 16.8	65.7 ± 10.6	0.011
lvEF, %		52.2 ± 11	63.1 ± 5.9	< 0.001
rvESV/BSA, mL/m^2^		31.8 ± 10.3	31 ± 11	0.758
rvEDV/BSA, mL/m^2^		70.8 ± 15.2	73 ± 14.7	0.535
rvEF, %		55.1 ± 8.7	58 ± 8.1	0.141
laEDV, mL//m^2^		63.4 ± 22.3	59.7 ± 18.3	0.058
laS_r, %		19.3 ± 2.9	24 ± 2.8	< 0.001
laS_cd, %		8.7 ± 3.4	12.5 ± 2.6	< 0.001
laS_bp, %		10.8 ± 2.6	9.7 ± 1.9	0.041
laSR_r, L/s		0.8 ± 0.2	1.2 ± 0.4	< 0.001
laSR_cd°, L/s (median [IQR])		-0.7, (-0.7, -0.3)	-1.1, (-2.0, -0.6)	< 0.001
laSR_bp, L/s		-1.1 ± 0.3	-1.3 ± 0.5	0.032
laGRS, %		36.3 ± 11.9	52.8 ± 9.8	< 0.001
lvGRS, %		25.2 ± 7.7	33.6 ± 8.2	0.005
lvGCS, %		-15.5 ± 3.5	-21.8 ± 2.9	< 0.001
lvGLS, %		-14 ± 4.1	-19.7 ± 2.6	< 0.001
lvGRSR, L/s		1.2 ± 0.3	1.6 ± 0.3	0.002
lvGCSR°, L/s		-0.8, (-1.3, -0.9)	-1.0, (-1.2, -0.8)	< 0.001
lvGLSR, L/s		-0.7 ± 0.2	-1.2 ± 0.9	0.001
lvEdema, n (%)		50 (100)	0 (0)	< 0.001
lvLGE, n (%)		3 (6)	0 (0)	0.892

Data are reported as mean ± standard deviation unless differently indicated

*BSA* Body surface area, *CMR* Cardiac magnetic resonance, *cTTS* Convalescence phase takotsubo syndrome, *EDV* End-diastolic volume, *EF* Ejection fraction, *ESV* End-systolic volume, *GCS* Global circumferential strain, *GCSR* Global circumferential strain rate, *GLS* Global longitudinal strain, *GLSR* Global longitudinal strain rate, *GRS* Global radial strain, *GRSR* Global radial strain rate, *la* Left atrial, *laS_bp* Left atrial booster pump strain, *laSR_bp* Left atrial booster pump strain rate, *laS_cd* Left atrial conduit strain, *laSR_cd* Left atrial conduit strain rate, *laS_r* Left atrial reservoir strain, *laSR_r* Left atrial reservoir strain rate, *LGE* Late gadolinium enhancement, *lv* Left ventricular, *sTTS* Subacute phase takotsubo syndrome

**Table 2:** Clinical data and CMR parameters of the study population (sTTs, cTTS, and controls)

Parameters		sTTS	cTTS	Controls	*p*-value
**Population,** ***n***		19	31	30	-
**Age, years**		67 ± 14	63 ± 11	60 ± 11	0.111
**Onset-to-CMR, days**		5.3 ± 2	14.2 ± 6	−	0.001
**Phenotype,** ***n (%)***	Apical	11 (58)	25 (81)	−	0.167
	Mid	5 (26)	3 (10)	−	0.171
	Basal	1 (5)	1 (3)	−	0.694
	Focal	2 (11)	2 (7)	−	0.576
**lvEF range,** ***n*** **(%)**	< 40%	3 (16)	3 (10)	−	0.103
	40–50%	6 (32)	4 (13)	−	0.001
	51–60%	7 (37)	14 (45)	8 (26)	0.323
	> 60%	3 (16)	10 (32)	23 (74)	< 0.001
**lvESV/BSA, mL/m**^**2**^		40.6 ± 18.4	34.4 ± 14.1	23.9 ± 9.6	0.208
**lvEDV/BSA, mL/m**^**2**^		76.3 ± 19	73.8 ± 15.8	65.7 ± 10.6	0.634
**lvEF, %**		47.4 ± 11.9	54.8 ± 9.9	63.1 ± 5.9	0.028**
**rvESV/BSA, mL/m**^**2**^		33.3 ± 9.2	31 ± 11	31 ± 11	0.486
**rvEDV/BSA, mL/m**^**2**^		70.2 ± 15.4	71.2 ± 15.3	73 ± 14.7	0.832
**rvEF, %**		52.7 ± 7.1	56.4 ± 9.2	58 ± 8.1	0.165
**laEDV, mL**		63.8 ± 19.8	64.9 ± 25.2	59.7 ± 18.3	0.553
**laS_r, %**		18.9 ± 2.7	19.5 ± 3	24 ± 2.8	< 0.001
**laS_cd, %**		7 ± 2.6	9.5 ± 3.5	12.5 ± 2.6	< 0.001**
**laS_bp, %**		12.7 ± 2.6	9.8 ± 2	9.7 ± 1.9	0.001
**laSR_r, l/s**		0.8 ± 2	0.8 ± 0.3	1.2 ± 0.4	< 0.001
**laSR_cd, l/s (median [iqr])**		-0.7, (-1.2, -0.3)	-0.7, (-1.8, -0.3)	-1.1, (-2.0, -0.6)	< 0.001
**laSR_bp, l/s**		-1.1 ± 0.3	-1 ± 0.3	-1.3 ± 0.5	0.078
**laGRS, %**		35.6 ± 11.4	36.7 ± 12.3	52.8 ± 9.8	0.001
**lvGRS, %**		23.2 ± 8.5	26.1 ± 7.2	33.6 ± 8.2	0.003
**lvGCS, %**		-14.5 ± 3.8	-16 ± 3.3	-21.8 ± 2.9	< 0.001
**lvGLS, %**		-12.2 ± 3.9	-14.6 ± 3.8	-19.7 ± 2.6	< 0.001**
**lvGRSR, l/s**		1.1 ± 0.3	1.3 ± 0.3	1.6 ± 0.3	0.001
**lvGCSR, l/s (median [iqr])**		-0.7, (-1.1, -0.4)	-0.8, (-1.26, -0.9)	-1.0, (-1.2, -0.7)	< 0.001
**lvGLSR, l/s**		-0.6 ± 0.2	-0.7 ± 0.2	-1.2 ± 0.6	< 0.001
**lvEDEMA, *n* (%)**		16 (100)	31 (100)	0 (0)	< 0.001
**lvLGE, *n* (%)**		1 (3)	2 (7)	0 (0)	0.369

Data are reported as mean ± standard deviation unless differently indicated

*BSA* Body surface area, *CMR* Cardiac magnetic resonance, *cTTS* Convalescence phase takotsubo syndrome, *EDV* End-diastolic volume, *EF* Ejection fraction, *ESV* End-systolic volume, *GCS* Global circumferential strain, *GCSR* Global circumferential strain rate, *GLS* Global longitudinal strain, *GLSR* Global longitudinal strain rate, *GRS* Global radial strain, *GRSR* Global radial strain rate, *la* Left atrial, *laS_bp* Left atrial booster pump strain, *laSR_bp* Left atrial booster pump strain rate, *laS_cd* Left atrial conduit strain, *laSR_cd* Left atrial conduit strain rate, *laS_r* Left atrial reservoir strain, *laSR_r* Left atrial reservoir strain rate, *LGE* Late gadolinium enhancement, *lv* Left ventricular, *sTTS* Subacute phase takotsubo syndrome

**p-values < 0.05 for all the comparisons (one-way ANOVA and Bonferroni post hoc analysis)

### CMR features

CMR data are presented in Tables 1 and 2. All the patients showed LV myocardial edema on T2-STIR images, while only three patients showed LGE areas with nonischemic mid-wall patterns. Specifically, two patients showed an LGE area on the lateral LV wall at apical segments and one on the anterior and lateral walls on the basal planes.

### TTS *versus* controls

In the TTS group, the lvEF, indexed end-diastolic volume (lvEDV/BSA), and end-systolic volume (lvESV/BSA) were reduced compared to controls (*p* < 0.012 for all). Conversely, no significant differences were found in the right ventricular volumes (rvEDV, rvEDV/BSA, rvESV, rvESV/BSA) and rvEF (*p* > 0.140 for all). The laS_r, laS_cd, laS_bp, laSR_r, and laSR_bp were altered in TTS patients compared to controls (*p* < 0.042 for all), whereas the laSR_cd did not show significant differences between the two groups (*p* = 0.288). All the LV strain and strain rate values (lvGRS, lvGCS, lvGLS, lvGRSR, lvGCSR, lvGLSR) were significantly reduced in TTS patients (*p* < 0.037).

### aTTS *versus* cTTS

**Fig. 5:**
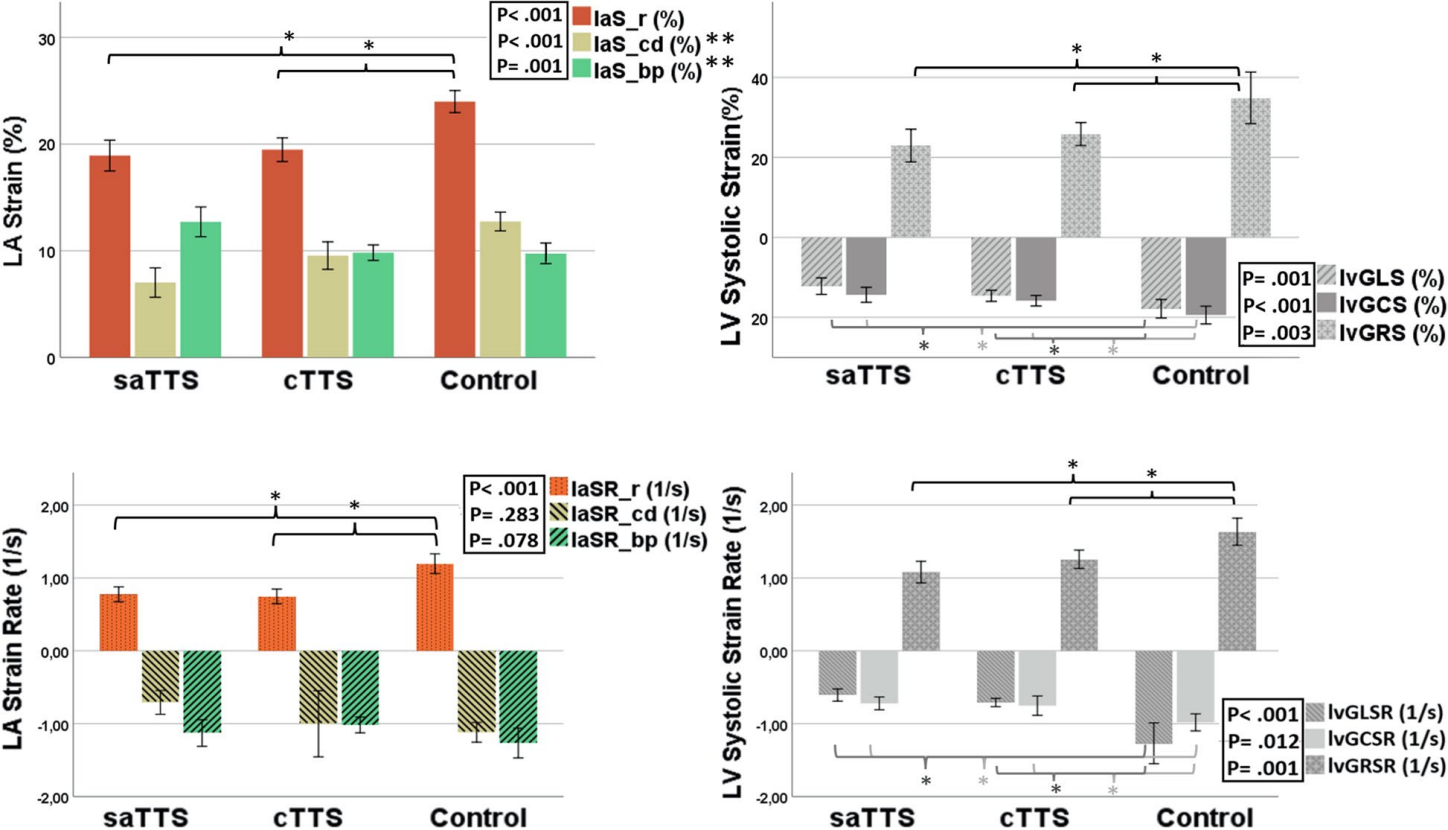
Histograms comparing mean values of LA and LV strain (**a**, **c**) and strain rate (**b**, **d**) in sTTS, cTTS, and control group (one-way ANOVA). *p-value < 0.05; **p-value < 0.05 for all the comparisons. *cTTS* Convalescence phase takotsubo syndrome, *LA* Left atrial, *laS_bp* LA booster pump strain, *laS_cd* LA conduit strain, *laS_r* LA reservoir strain, *LV* left ventricular, *lvGCS* LV global circumferential strain, *lvGLS* LVglobal longitudinal strain, lvGRS LV global radial strain, *SR* Strain rate, *sTTS* Subacute phase takotsubo syndrome

The sTTS and cTTS patients did not show any differences in age and phenotype prevalence (*p* > 0.110 for all). The LV- and RV-EDV, ESV, EDV/BSA, and ESV/BSA were comparable between the sTTS and cTTS (*p* > 0.163 for all). The lvEF mean value resulted lower in sTTS than cTTS (47.4 ± 11.9 *versus* 54.8 ± 9.9; *p* = 0.010). The rvEF was normal in the two groups without any significant differences (*p* = 0.076). As shown in Fig. 5, sTTS demonstrated laS_cd (7 ± 2.6 *versus* 9.5 ± 3.5; *p* = 0.004), and laS_bp (12.7 ± 2.6 *versus* 9.8 ± 2.0; *p* < 0.001) significantly reduced if compared to cTTS; conversely, the laS_r was comparable between the sTTS and cTTS (18.9 ± 2.7 *versus* 19.5 ± 3.0; *p* = 0.503). None of the laSR values showed significant differences between the two TTS subgroups (*p* > 0.244 for all).

The lvGLS was reduced in sTTS (-12.1 ± 3.6 *versus* -14.6 ± 3.8; *p* = 0.029) compared to cTTS, whereas lvGCS and lvGRS did not show any statistical differences (*p* = 0.301 and *p* = 0.484, respectively).

### Association between LA and LV function and onset-to-CMR time

**Fig. 6:**
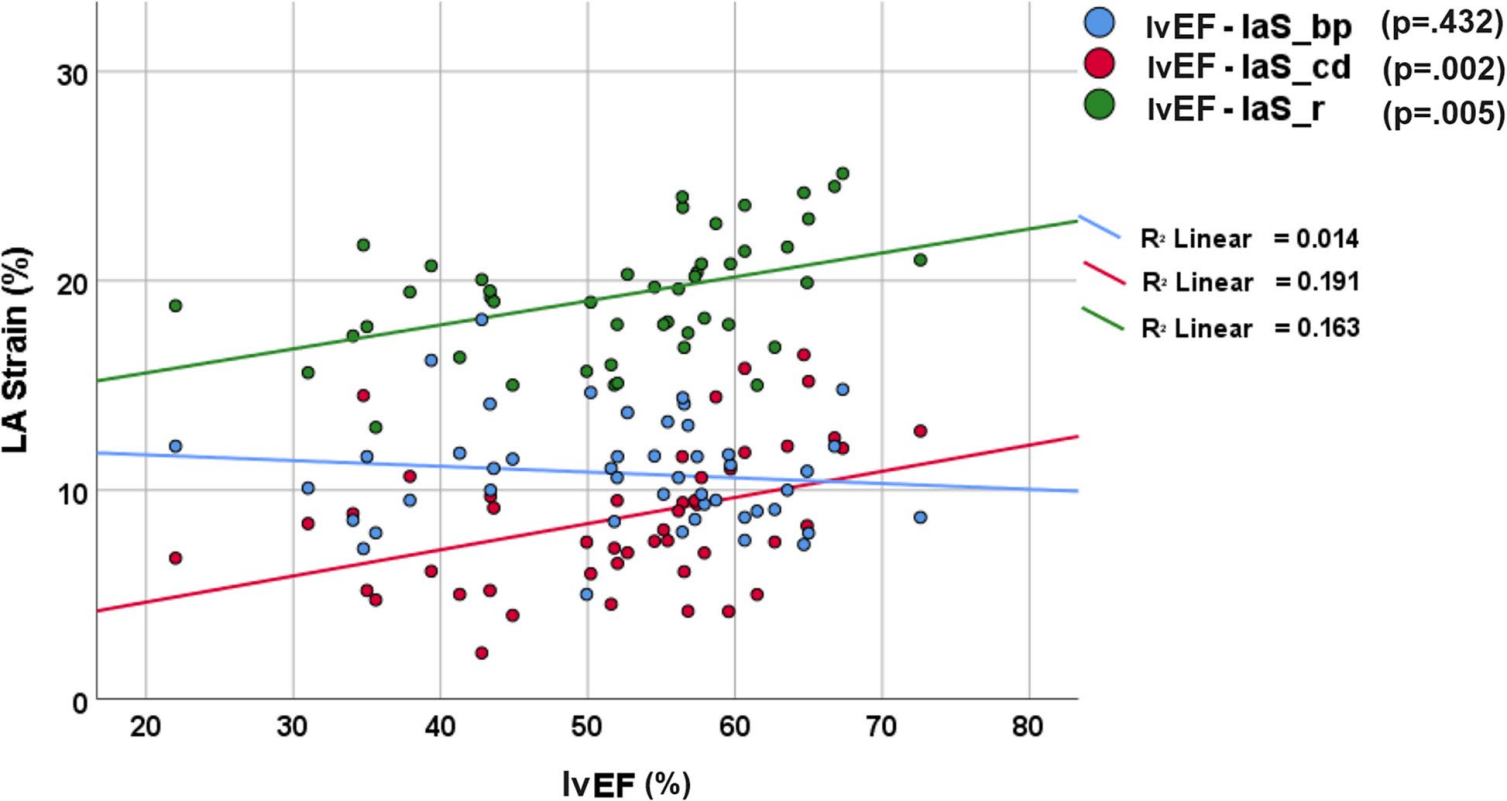
Scatter plot and adaptation lines showing the trend of the laS_r, laS_cd, and laS_bp according to the LV-EF. *laS_cd* Left atrial conduit strain, *laS_r* Left atrial reservoir strain, *LV-EF* Left ventricular ejection fraction

Scatter plot graph with linear regression analysis between lvEF and LA strain values is shown in Fig. 6. Both the laS_r and laS_cd showed a moderate direct linear correlation with the lvEF (*r* = 0.404, *p* = 0.005 and *r* = 0.437, *p* = 0.002, respectively). No correlations were found between the lvEF and laS_bp or laSR parameters (*p* > 0.149).

**Fig. 7:**
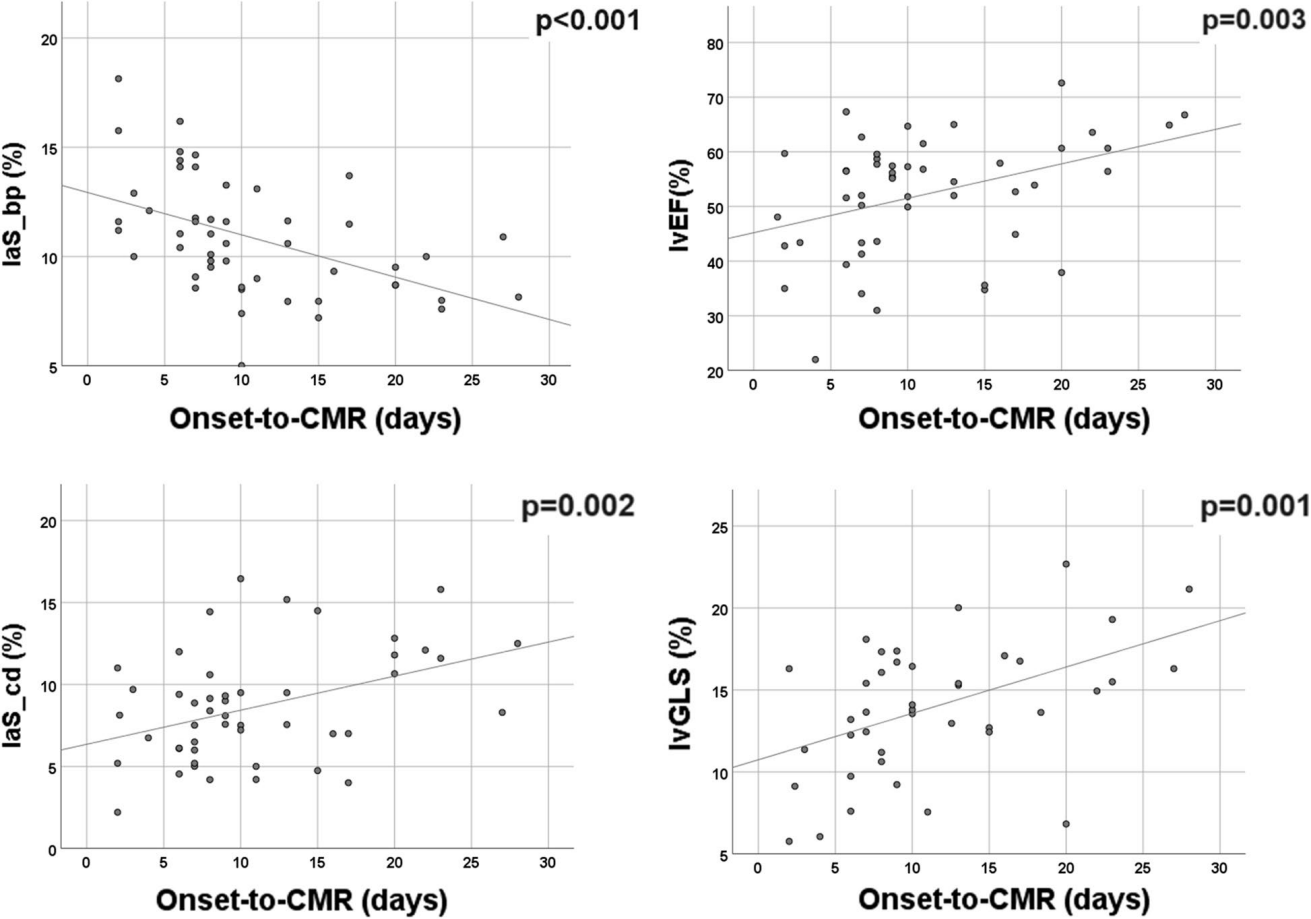
Adaptation line and scatter plots showing the distribution of left atrial booster pump (**a**) and conduit (**b**) strain, lvEF (**c**), and lvGLS (**d**), according to the time elapsed since symptomatology onset and CMR (cardiac magnetic resonance). *laS_bp* Left atrial booster pump strain, *laS_cd* Left atrial conduit strain, *lvEF* Left ventricular ejection fraction, *lvGLS* Left ventricular global longitudinal strain

Possible associations between LA strain values and the onset-to-CMR time were investigated. Lower laS_cd and higher laS_bp were found in sTTS subgroup compared to the cTTS (Table 2), associated with a progressive increase of laS_cd and decrease of laS_bp as the days passed after the symptoms onset (Fig. 7). Accordingly, laS_bp (*r* = -0.484, *p* = 0.001), laS_cd (*r* = 0.398, *p* = 0.002), lvGLS (*r* = 0.470, *p* = 0.001), and lvEF (*r* = 0.374, *p* = 0.003) showed a moderate to fair correlation with the interval between symptoms onset and the CMR. Conversely, no significant differences were found in laS_r between sTTS and cTTS (Table 2).

None of the laSR parameters was found to be correlated with the phase of pathology (*p* > 0.354).

Assessing the relationship between the laS_bp and laS_cd, and onset-to-CMR time, we conducted a comprehensive linear regression analysis that confirmed the interdependence between the variables (*p* = 0.002 and 0.008, beta = -0.424 and 0.356, respectively).

The age (*p* = 0.012 and 0.003, beta = 0.337 and -0.406, respectively) was inserted in the model confirming the previous result for both the atrial strain parameters (*r* = 0.549 and 0.547, respectively) with a mildly better correlation for the laS_bp.

### Strain parameters as markers of temporality

**Fig. 8:**
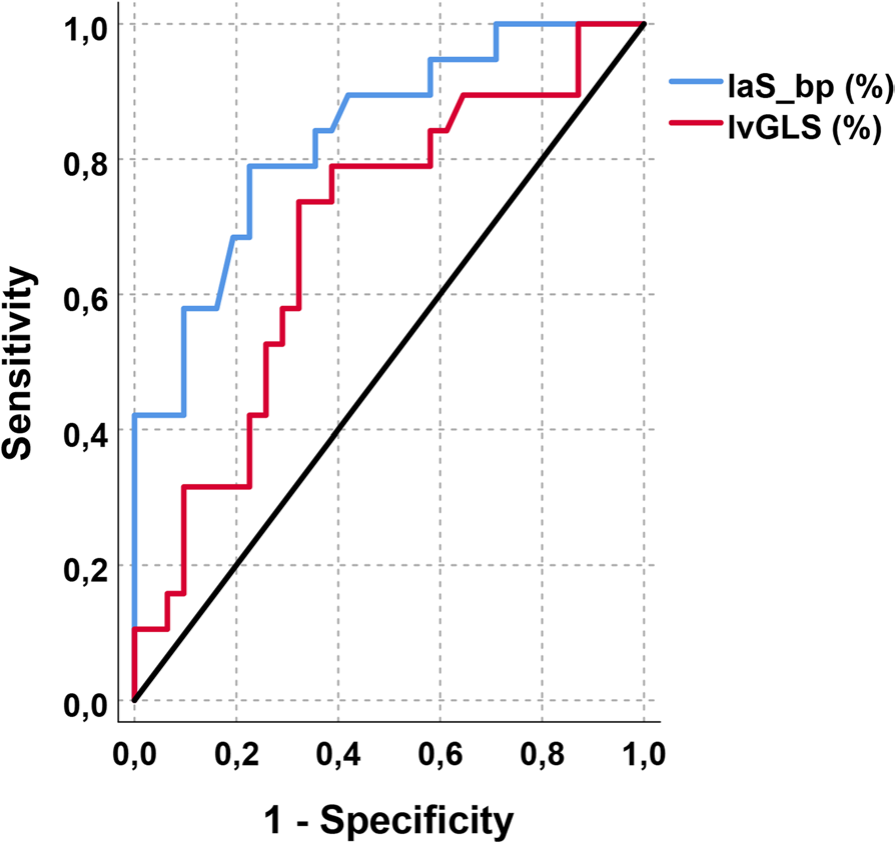
Receiver operator characteristics analysis of the laS_bp (blue) and lvGLS (red) for identifying the sTTS. laS_bp (AUC 0.815, 95% confidence interval 0.684 − 0.945, *p* < 0.001) and lvGLS (AUC 0.670, confidence interval 0.506 − 0.835, *p* = 0.043). *AUC* Area under the curve, *laS_bp* Left atrial booster pump strain, *laS_cd* Left atrial conduit strain, *lvEF* Left ventricular ejection fraction, *lvGLS* Left ventricular global longitudinal strain

We assessed the capability of LA and LV strain values to distinguish between subacute and convalescent TTS phases using ROC analysis. lvEF and laS_cd failed to discriminate between the sTTS and cTTS (area under the curve [AUC] < 0.288, *p* = 0.001, for both), whereas laS_bp proved to have an excellent discriminatory power (AUC = 0.815, *p* < 0.001), followed by lvGLS (AUC = 0.670; *p* = 0.043), as shown in Fig. 8.

The best cutoffs for the distinction between cTTS and cTTS groups were 11% for laS_bp (81% sensitivity and 74% specificity) and 13.4% for the lvGLS (69% and 68%, respectively).

laS_r and laS_bp showed good to excellent intra- and inter-observer reproducibility (ICC 0.74–0.91, *p* < 0.001) without any significant systematic bias, whereas laS_cd demonstrated only a fair to good reproducibility (ICC 0.48–0.55, *p* = 0.049); Supplementary material, Table S1).

## Discussion

There is growing evidence that left atrium plays a central role in cardiovascular physiology, serving as reservoir, conduit, and booster pump for efficient cardiac function and blood flow [28]. Moreover, the quantitative assessment of LA strain has proven to be a superior prognostic marker compared to other echocardiographic parameters in many cardiovascular diseases [29–32], including atrial fibrillation, heart failure, and stroke [33, 34].

Our study contributes to implementing the knowledge about changes in ventricular and atrial function in patients with TTS using myocardial strain analysis assessed by CMR feature tracking. The main findings of our study can be summarized as follows: (i) TTS leads to impairment of LA function, which persists for weeks after the onset of symptoms, even when ventricular function is restored, (ii) atrial strain parameters change in the weeks following the acute episode, and (iii) the laS_bp is the best discriminator between subacute and convalescent phases and could represent a good marker of TTS healing, even better than the LV strain.

### Ventricular strain

It is known that during TTS, the lvEF can be only mildly to moderately reduced since the hypercontractility of noninvolved regions balances the pronounced contractile impairment of the affected segments. Furthermore, it is not uncommon for the lvEF to normalize already in the subacute phase. For these reasons, lvEF should not be considered an adequate marker of LV dysfunction [35], showing only limited prognostic value [36]. Indeed, in our population, the lvEF was mildly reduced in sTTS and at the lower limit of the normal range in cTTS [37].

Ventricular myocardial strain analysis could allow for a better and more accurate definition of systolic dysfunction. In fact, all ventricular strain parameters (lvGCS, lvGRS, and lvGLS) were altered when compared with controls, and lvGLS was the only parameter showing a significant difference in the comparison between subgroups (sTTS *versus* cTTS), being reduced in sTTS. The impairment of all ventricular strain components suggests that the myocardial injury is transmural and affects all layers of the ventricular wall [38], even if the lvGLS seems to be the most compromised parameter in our population, consistent with possible greater damage to the subendocardial myocardium. We also found that lvGLS could be a good marker of sTTS at ROC analysis.

### Atrial strain

According to our results, the impairment of atrial function persists longer after the symptoms onset (even up to a month), regardless of the restoration of LV systolic function, and this should be considered a distinct and peculiar feature of TTS [19], especially for the subacute phase. This result is probably the combination of a direct insult on the atrial wall, mediated by high levels of circulating catecholamines [39] and an adenosine monophosphate-mediated calcium overload [40, 41], and the LV diastolic dysfunction, which increases the filling pressures and the stiffness, causing an imbalance of atrioventricular coupling [42].

In addition, the comparison between sTTS and cTTS revealed peculiar differences in reservoir, conduit, and booster pump functions. LaS_r takes part in the atrial response to the early stage of LV filling [43] and is related to LV compliance [28, 44]. According to the literature, LaS_r was reported to be a marker of TTS acute phase [19, 45] and a predictor of in-hospital outcomes [46, 47]. In our study, this parameter was impaired in both subgroups, with no differences between sTTS and cTTS. Conversely, the conduit strain was impaired in the sTTS subgroup only, with a progressive improvement during the later phase, and its modification was directly correlated to the lvEF and LV strain values. These results are in accordance with a previous study by Backhaus et al. [19], which revealed a reduction in laS_cd during the acute phase of TTS and a significant increase at the follow-up. LaS_cd is generally reduced in conditions associated with ventricular diastolic dysfunction [43]. Indeed, it relies on atrial compliance during ventricular diastole and is closely related to LV relaxation and stiffness [48–50].

Finally, the LA booster pump function was significantly increased in TTS compared to controls, with peak values reached during the subacute phase, gradually decreasing during the convalescent phase.

The booster pump represents the intrinsic atrial contractility, depending on venous return, left ventricular diastolic compliance, and pressure [48]. Its increased function is a known compensatory mechanism when diastolic ventricular dysfunction occurs [51]. This mechanism was already demonstrated during the acute phase of TTS [19], and, in our population, it persisted during the subacute, but it tends to resolve during convalescence.

In our cohort, the laS_bp was the most sensitive and specific imaging marker of sTTS (AUC: 0.815; *Se*: 81% and *Sp*: 74%), in discriminating between subacute and convalescent phases, performing even better than the lvGLS (AUC: 0.670) and independently by lvEF and LV strain parameters. This result suggests that in sTTS, a residual mild diastolic dysfunction prevails over the systolic one, and it can be precisely measured by the laS_bp.

Beyond speculations on the role of atrial function in the pathophysiology, the laS_bp could represent a useful marker in temporal determination and risk stratification among TTS patients. In previous studies, the LA active contraction was able to characterize early-stage LV filling impairment [43] and demonstrated to be a useful prognostic marker in some cardiac conditions. In patients with heart failure and preserved [52] or reduced EF [20, 44], LA booster pump was an incremental predictor of life-threatening ventricular arrhythmias in nonischemic cardiomyopathy [53] and a potential predictor of postoperative atrial fibrillation in patients with severe aortic stenosis [54]. In the TTS setting, laS_bp showed a good performance in discriminating between low- and high-risk groups regarding adverse clinical events [19] and demonstrated an association with mortality, even after correction for age. Therefore, it could be a useful tool in recognizing patients with incomplete or delayed functional recovery, who might be at greater risk of events and would require the optimization of medical therapy.

Implementing the comprehensive assessment of tissue and functional abnormalities offered by CMR with the quantitative analysis of LA and LV strains could improve the risk stratification of these patients and the tailoring of patient-targeted therapies.

This study has limitations. First, the population under analysis is numerically limited and composed exclusively of women. The results obtained should be verified in multicentric studies with larger populations, and different equipment, since these data may have been influenced by the type of scanner and software analysis. Second, the subjects included in the control group underwent CMR for the following indications: suspected LV noncompaction cardiomyopathy at echocardiography (not confirmed by CMR), isolated ventricular extrasystoles, and cardiac pseudomasses. Therefore, subtle abnormalities in atrial or ventricular strain parameters cannot be excluded with certainty. Third, patients with poor diagnostic quality, frequently with worse clinical conditions, were excluded from the study, as well as unstable patients, who did not undergo CMR. Fourth, the time intervals for the classification of TTS patients in the subacute and convalescent phases were arbitrary and consistent with the available literature; however, it brings an inevitable generalization, and could not reflect the effective individual clinical evolution of the disease.

Fifth, the lack of CMR exams performed before the TTS or at long-term follow-up does not allow us to exclude that the described alterations were, in some patients, preexisting at the onset of TTS and not associated with its occurrence. Sixth, only the longitudinal and radial atrial strain have been measured due to the availability in all patients of cineMR images acquired only in the long axis (cineMR images acquired in the short axis, covering LA were not systematically acquired and therefore were not used for the measurement of circumferential strain). Seventh, the values of the end-diastolic filling pressures of the left ventricle are missing. The evaluation of this parameter and its relationships with atrial strain could help to better understand the alterations in left atrial function, in particular those concerning the booster pump.

In conclusion, LA dysfunction persisted during the subacute and convalescent phases of TTS. In particular, the booster pump component of LA function increased in the subacute phase and showed a progressive decrease during the convalescence, independent of the LV function (EF and GLS). LaS_bp was the best discriminator between patients with TTS in subacute and convalescent phases and could represent a useful index of functional recovery. Atrial strain parameters can improve the characterization of cardiac injury and functional impairment in TTS, aiding in the identification of high-risk patients and facilitating the implementation of more appropriate and tailored medical therapy.

### Supplementary Information

**Additional file 1: Supplementary Table 1.** Inter- and intra-reader reproducibility for left atrium strain measurement (*n* = 80) made with two-way mixed model and absolute agreement ICC.

## Data Availability

The dataset of the study is available from the corresponding author upon reasonable request.

## Abbreviations

AUC: Area under the curve
BSA: Body surface area
CMR: Cardiac magnetic resonance
cTTS: Convalescence phase takotsubo syndrome
EDV: End-diastolic volume
EF: Ejection fraction
ESV: End-systolic volume
GLS: Global longitudinal strain
GLSR: Global longitudinal strain rate
GRSR: Global radial strain rate
ICC: Intraclass correlation coefficient
LA: Left atrial
laEDV: Left atrial end-diastolic volume
laS_bp: Left atrial booster pump strain
laS_cd: Left atrial conduit strain
laS_r: Left atrial reservoir strain
laSR_bp: Left atrial booster pump strain rate
laSR_cd: Left atrial conduit strain rate
laSR_r: Left atrial reservoir strain rate
LGE: Late gadolinium enhancement.
LV: Left ventricular
ROC: Receiver operator characteristics
sTTS: Subacute phase takotsubo syndrome
T2-STIR: T2-weighted short tau inversion-recovery
TTS: Takotsubo syndrome
