# Supplementary material for: Takotsubo syndrome: left atrial and ventricular myocardial strain impairment in the subacute and convalescent phases assessed by CMR
Source: Eur Radiol Exp. 2024 Feb 28;8:34. doi: 10.1186/s41747-024-00423-7 (PMC10899127; doi:10.1186/s41747-024-00423-7)
Supplement: Supplementary file 1 — Additional file 1: Supplementary Table 1. Inter- and intra-reader reproducibility for left atrium strain measurement (n = 80) made with two-way mixed model and absolute agreement ICC. [file 41747_2024_423_MOESM1_ESM.docx]

**Takotsubo syndrome: left atrial and ventricular myocardial strain impairment in the subacute and convalescent phases assessed by CMR**

**ELECTRONIC SUPPLEMENTARY MATERIAL**

**Supplementary Table 1** Inter- and intra-reader reproducibility for left atrium strain measurement (n = 80) made with two-way mixed model and absolute agreement ICC.

| **Inter-reader** | | | | **Intra-reader** | | |
| --- | --- | --- | --- | --- | --- | --- |
| **LA parameter** | **CI 95% Limits**  **(lower - upper)** | **ICC** | ***p*** | **CI 95% Limits**  **(lower - upper)** | **ICC** | ***p*** |
| laS_r (%) | 0.839 - 0.935 | 0.898 | <0.001 | 0.977 - 0.991 | 0.985 | <0.001 |
| laS_cd (%) | 0.845 - 0.936 | 0.900 | <0.05 | 0.980 - 0.992 | 0.987 | <0.001 |
| laS_bp (%) | 0.816 - 0.926 | 0.883 | 0.001 | 0.964 - 0.985 | 0.977 | <0.001 |

*CI confidence interval, ICC intraclass correlation coefficient, laS_bp left atrial booster pump strain, laS_r left atrial reservoir strain, laS_cd left atrial conduit strain.*
